# Nutritional Status in Nocturnal Hemodialysis Patients – A Systematic Review with Meta-Analysis

**DOI:** 10.1371/journal.pone.0157621

**Published:** 2016-06-20

**Authors:** Karin J. R. Ipema, Simone Struijk, Annet van der Velden, Ralf Westerhuis, Cees P. van der Schans, Carlo A. J. M. Gaillard, Wim P. Krijnen, Casper F. M. Franssen

**Affiliations:** 1 Dialysis Center Groningen, Groningen, The Netherlands; 2 Research group Healthy Ageing, Allied Health Care and Nursing, Hanze University Groningen, University of Applied Sciences, Groningen, the Netherlands; 3 Department of Internal Medicine, Division of Nephrology, University Medical Center Groningen, University of Groningen, Groningen, The Netherlands; 4 Department of Rehabilitation Medicine, Center for Rehabilitation, University Medical Center Groningen, University of Groningen, Groningen, The Netherlands; Hvidovre Hospital, DENMARK

## Abstract

**Background:**

Hemodialysis patients experience an elevated risk of malnutrition associated with increased morbidity and mortality. Nocturnal hemodialysis (NHD) results in more effective removal of waste products and fluids. Therefore, diet and fluid restrictions are less restricted in NHD patients. However, it is ambiguous whether transition from conventional hemodialysis (CHD) to NHD leads to improved intake and nutritional status. We studied the effect of NHD on protein intake, laboratory indices of nutritional status, and body composition.

**Study design:**

Systematic review with meta-analysis.

**Population:**

NHD patients.

**Search strategy:**

Systematic literature search from databases, Medline, Cinahl, EMBASE and The Cochrane Library, to identify studies reporting on nutritional status post-transition from CHD to NHD.

**Intervention:**

Transition from CHD to NHD.

**Outcomes:**

Albumin, normalized protein catabolic rate (nPCR), dry body weight (DBW), body mass index (BMI), phase angle, protein intake, and energy intake.

**Results:**

Systematic literature search revealed 13 studies comprising 282 patients that made the transition from CHD to NHD. Meta-analysis included nine studies in 229 patients. In control group controlled studies (n = 4), serum albumin increased significantly from baseline to 4–6 months in NHD patients compared with patients that remained on CHD (mean difference 1.3 g/l, 95% CI 0.02; 2.58, p = 0.05). In baseline controlled studies, from baseline to 4–6 months of NHD treatment, significant increases were ascertained in serum albumin (mean difference (MD) 1.63 g/l, 95% CI 0.73–2.53, p<0.001); nPCR (MD 0.16 g/kg/day; 95% CI 0.04–0.29, p = 0.01); protein intake (MD 18.9 g, 95% CI 9.7–28.2, p<0.001); and energy intake (MD 183.2 kcal, 95% CI 16.8–349.7, p = 0.03). Homogeneity was rejected only for nPCR (baseline versus 4–6 months). DBW, BMI, and phase angle did not significantly change. Similar results were obtained for comparison between baseline and 8–12 months of NHD treatment.

**Limitations:**

Most studies had moderate sample sizes; some had incomplete dietary records and relatively brief follow-up period. Studies markedly differed with regard to study design.

**Conclusions:**

NHD is associated with significantly higher protein and energy intake as well as increases in serum albumin and nPCR. However, the data on body composition are inconclusive.

## Introduction

Malnutrition is a frequent problem experienced by hemodialysis patients and forms one of the primary causes of the high morbidity and mortality in this patient group [1,2]. Various factors contribute to malnutrition in patients with end-stage renal disease, e.g., increased protein and energy requirements [3], reduced appetite and food-intake [1,4,5], protein-energy wasting as a result of chronic inflammation [6,7], and reduced physical activity [8]. Frequent nocturnal home hemodialysis (NHHD) is the dialysis treatment with the greatest weekly removal of uremic toxins. Nocturnal in-centre hemodialysis (NCHD) is an alternative for patients who are not applicable for home hemodialysis and have medical and/or social reasons for nocturnal or more efficient hemodialysis.

According to several studies, prolonged hemodialysis sessions, either at home or in-center, may improve clinical outcomes [9–11]. A hemodialysis schedule of three times per week of eight hours improved nutritional status, volume status, and survival rates [12]. Nocturnal hemodialysis (NHD) combines the long duration dialysis and more frequent treatments which results in ameliorated elimination of waste products and fluids. Therefore, diet and fluid restrictions are usually less stringent in NHD patients compared with patients on conventional hemodialysis (CHD). NHD patients use significantly less or no phosphate binders which may result in a revitalized appetite [13,14]. Receiving dialysis during the night may also facilitate a more 'normal' day rhythm with less disturbances of eating patterns. All of these factors may modify the pattern of food consumption after the transition from CHD to NHD.

Current knowledge regarding long-term effects of NHD on nutritional status is limited. Therefore, we performed a systematic review on the effect on nutritional status with the transition from CHD to NHD. We specifically studied the effect of NHD on protein intake, laboratory indices of nutritional status, and body composition.

## Methods

### Study protocol and information sources

This systematic review was performed between October 3^rd^, 2013, and February 1^st^, 2014, from databases of Medline, Cinahl, EMBASE, and the Cochrane Library, according study protocol (S1 File). We followed the PRISMA recommendations for reporting data and meta-analysis (PRISMA 2009) (S1 Table).

### Eligibility criteria

Studies were eligible for inclusion if the following criteria were satisfied: (1) nocturnal hemodialysis administered to adults (aged ≥18 years) experiencing chronic kidney disease; (2) outcomes of interest are scores relating to the patients nutritional status; and (3) full-length articles published between January 1^st^, 1990, and February 1^st^, 2014, and written in English. Data was required to be obtained by original research and not from reviews. Due to an expected minimal number of randomized controlled trials (RCT’s), there was no exclusion criterion based upon methodology applied to the studies. The selection procedure included RCT's, controlled trials, cohort studies including observational studies with a comparison group, case-control studies, and prospective longitudinal studies comparing nutritional parameters prior to and following the transition from CHD to NHD.

### Search strategy

Different combinations of terms and ‘strings’ were exploited in order to examine the effects of the search strategy. The search strategy for Medline is detailed in Table 1.

**Table 1:** Search strategy for Medline.

**To locate hemodialysis:**
1. Renal Dialysis [Mesh:noexp]
2. Hemodialysis, Home [Mesh]
3. Hemodialysis* [tw]
4. Haemodialysis*[tw]
5. Or/1-4
**To locate Nocturnal**
6. Nocturnal [tw]
7. Night* [tw]
8. Or/6-7
**To locate Nutritional status**
9. Malnutrition [Mesh]
10. Nutritional Physiological Phenomena [Mesh]
11. Body weights and Measures [Mesh]
12. Nutrition [tw]
13. Food* [tw]
14. Protein [tw]
15. Proteins [tw]
16. Energy [tw]
17. Body composition [tw]
18. Or/9-17
19. 5 and 8 and 18

### Study selection and data collection

Two reviewers (SS and AV) separately screened the titles and abstracts of the studies that were identified through electronic searching in order to select those that were potentially eligible for inclusion. Additional studies were identified through the references of the pertinent studies. After the screening, the reviewers discussed any variances in the study selection. Studies were determined as definitely eligible for inclusion if outcomes were available for laboratory measurements, food intake, or body composition in NHD patients. Study details (publication year, study design, patients’ characteristics, interventions, and outcomes) were aggregated in a standard spreadsheet (Microsoft Corporation, Redmond, Washington, USA) for summary and analyses.

### Risk of bias and quality assessment

All full-text versions of potentially relevant studies were independently screened by three reviewers (SS, AV, KI) to identify whether studies were definitely eligible for inclusion and to assess their quality. Study quality was assessed by employing the Newcastle-Ottawa Scale for cohort studies [15]. The scale consists of three quality criteria: selection, comparability, and outcome. The maximal score is 9 points (4 for selection, 2 for comparability, and 3 for outcome). Study quality was defined as poor when the score was 1–3, fair when the score was 4–6, and good when the score was 7–9 points.

### Data extraction

Information was collected of study designs, participant details, exclusion and inclusion criteria, interventions, and any comparators and outcomes. The following variables were selected after a pilot search and included as outcome variables: dry body weight, pre-dialysis weight, post-dialysis weight, dry lean body mass, interdialytic weight gain, fat mass, body mass index (BMI), body cell mass (BCM), extracellular mass (ECM), extracellular water (ECW), intracellular water (ICW), energy intake, protein intake, carbohydrate intake, fat intake, albumin levels, (normalized) protein catabolic rate ((n)PCR), (normalized) protein nitrogen appearance ((n)PNA), C-Reactive protein (CRP), bioimpedance phase angle, mid upper arm muscle circumference (MUAMC), arm muscle area.

### Statistical analyses

To assess differences in nutritional parameters, albumin, nPCR, dry body weight, BMI, phase angle, protein intake, and energy intake between baseline (on CHD) and, after a certain duration on NHD, a meta-analysis with a calculation of the mean differences with a 95% confidence interval was performed utilizing statistical programming language R (R Development Core Team (2014). There were two types of studies: ‘baseline controlled studies’ in which parameters at baseline and after a certain period on NHD were compared and ‘control group controlled’ studies that compared patients who made the transition from CHD to NHD with a control-group of patients that remained on CHD. For meta-analyses, data from baseline controlled and the NHD-arm of control group controlled studies were pooled whenever possible. To assess the differences in albumin between the NHD group and the control group, we also performed a meta-analysis (other nutritional parameters were assessed in too few control group controlled studies), with a calculation of the differences in mean with a 95% confidence interval.

Meta-analyses were conducted when the results of this parameter were available in three or more studies. Studies varied during the intervals wherein nutritional parameters were assessed after the transition from CHD to NHD. For surveying feasibility, we combined assessments for 4 to 6 months and for 8 to 12 months following the transition. The weights of the meta-analyses were based on the inverse variance method; the heterogeneity parameter (Tau^2^) for effect size was based on restricted maximum-likelihood. The random effects model was selected for the mean difference because an omnipresent treatment effect not being identical in size across all of the studies was expected [16]. Forest plots were constructed to summarize the outcome of the meta-analyses.

Characteristics of the NHD patients and the control groups as described in the studies were reported as mean±SD. One study reported the standard error (SE) as opposed to standard deviation (SD) [14]. The original data were kindly provided to us by the authors and, consecutively, the SD was calculated from the SE and the number of patients (n) as SD = SE*√n. One study had two research groups receiving nocturnal HD; one group dialyzed three times per week, and the other group dialyzed six times per week [17]. The data of these two research groups were included separately in the meta-analysis. In the meta-analysis for the comparison of NHD patients and controls, we primarily included the NHD group that dialyzed six times per week. In a separate analysis, we also performed the meta-analyses employing the NHD group that dialyzed three times per week instead of the group that dialyzed six times per week. This study incorporated two control groups with day-time hemodialysis: one group receiving dialysis three times per week for 2.5 to 4.0 hours and one group having dialysis six times per week for 1.5 to 2.75 hours. In our analyses, we only used the control group of three times per week HD because this dialysis schedule was most appropriately comparable with other studies.

Kaysen et al reported the ePCR while the majority of the other studies reported the nPCR [17]. Upon our request, the authors provided the results of the nPCR [17]. Since one study did not report the results of (the change in) dry body weight in the text, these results were estimated from the figure [18].

An influence analysis was a component of each meta-analysis to check whether the conclusion critically depends upon the results of a single study. A test for funnel plot asymmetry based on the linear regression method was added to indicate any risk of bias. Only funnel plots containing outlying studies are shown in order to save space. P-values <0.05 were considered statistically significant.

## Results

### Search results

**Fig 1:**
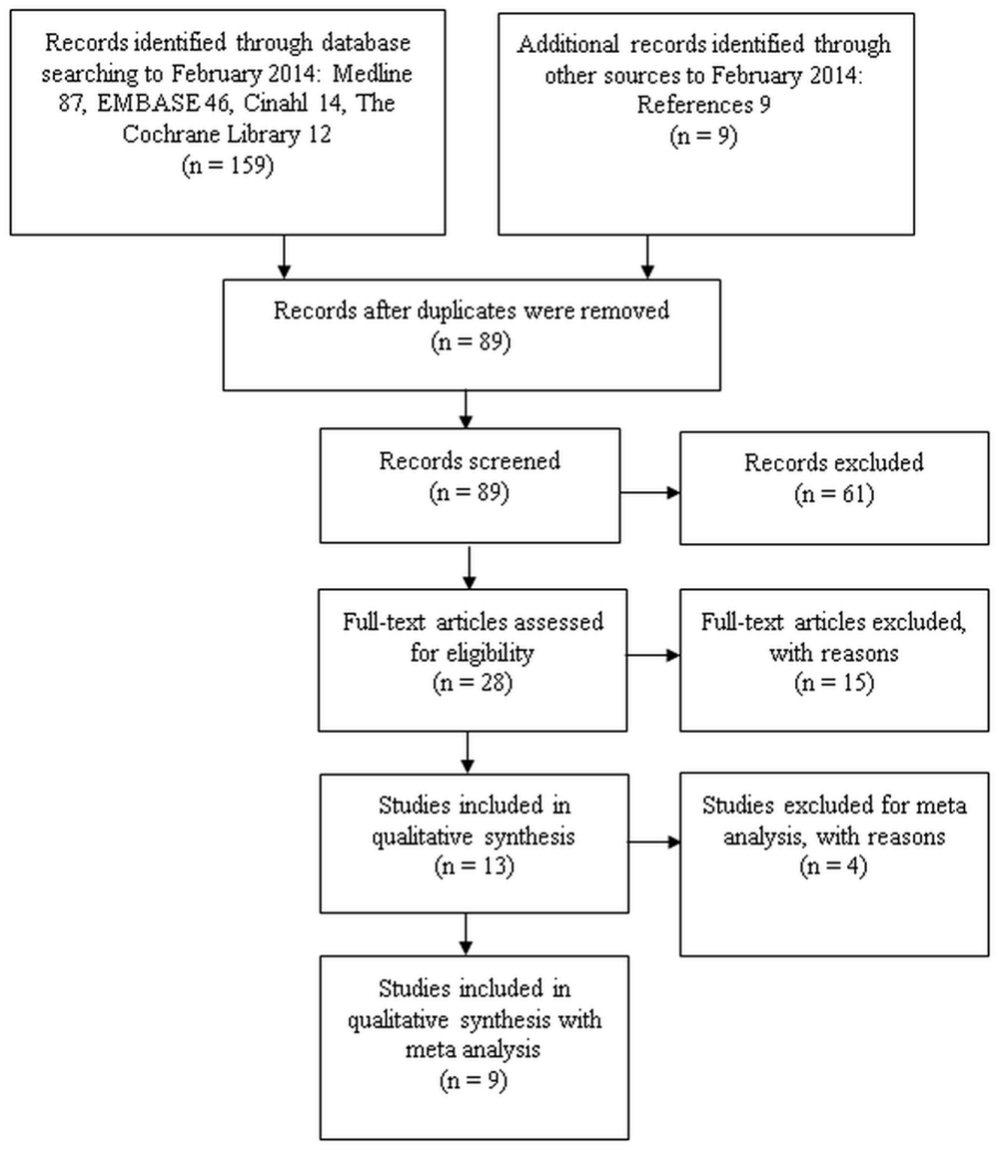
Selection of articles for the systematic review on nutritional status in NHD patients.

The literature search yielded a total of 563 articles: 156 in Medline, 300 in EMBASE, 40 in Cinahl, and 67 in the Cochrane Library. After screening the titles (of these 563 articles) 159 manuscripts and 9 references remained from other sources such as searching citations and examination of reference lists from relevant studies. After eliminating the duplicates, 89 records remained. The abstracts of these articles were screened by 3 reviewers (SS, AV, KI). A total of 28 full-text articles were assessed for eligibility after which 15 full-text articles were excluded as they did not meet the inclusion criteria for original research [19–25] or for other reasons, e.g., the focus on phosphate control [26–29], survival [10,30], dialysis efficiency [31], and left ventricular mass [32]. Finally, 13 studies were included for this systematic review (Fig 1). Of these, four studies were excluded from meta-analysis for different reasons. In one study, merely five participants were studied with an observation period of only eight weeks [33], in one study, no SD’s were available [34]; in another study, the duration on NHD was not clearly stated [35]. An additional study was excluded because nocturnal hemodialysis was performed with online hemodiafiltration [36]. The remaining nine studies incorporated data on one or more nutritional parameters, and these studies were included in the meta-analysis of the effect of nocturnal hemodialysis on nutritional status (Fig 1).

### Study designs, characteristics, and participants

Table 2 indicates study designs, study characteristics, information on participants, and study quality. Detailed results of biochemical nutritional parameters, body composition, and food records are summarized in S2 Table.

**Table 2:** Summary of characteristics studies.

Author	Duration of studies (months)	No. NHD patients	Men (n)	Mean age (years)mean±SD	In-centre /home NHD	Duration of NHD treatment	Study design	Single centre y/n	Control group y/n	Meta analysisy/n	Study quality
Alloatti,2002	>6	13	12 (92.3%)	52.0±13.0	In-centre	3 times/week for 8 hours	prospective	Y	N	N	4
Cravedi,2009	24	7	6 (85.7%)	50.4±11.0	In-centre	3 times/week for 8 hours	retrospective	Y	N	Y	5
David,2009	12	13	11 (84.6%)	34.8±13.7	In-centre	3 times/week for 8 hours	prospectivelongitudinal	Y	N	Y	5
Demirci,2013	12	57	41 (71.9%)	47.1±11.7	In-centre	3 times/week for 8 hours	prospective cohort trial	N	Y	Y	7
Ipema,2012	8	15	11 (73.3%)	53.4±10.3	Home	5/6 times/week for 8 hours	prospective observational	Y	N	Y	5
Kaysen,2012	12	87	57 (65.5%)		Home		prospective randomized trial	N	Y	Y	8
		42	28 (66.7%)	54.0±12.9		3 times/week ≥ 2.5 hours					
		45	29 (64.4%)	51.7±14.4		6 times/week ≥ 6 hours					
Maduel,2011	12	26	18 (69.2%)	49.2±14.0	In-centre		crossover prospective	Y	N	N	8
McPhatter,1999	18	11	5 (45.5%)	50.0±na	Home	5–6 times/week for 4–9 hours	prospectiveobservational	Y	N	N	5
O’Sullivan, 1998	2	5	3 (60%)	46,6±na	In-centre	6 times/week for 8 hours	pilot study	Y	N	N	3
Pierratos,1997	36	11	8 (72.7%)	40±10.0	Home	6–7 times/week for 8–10 hours	prospective	Y	N	Y	5
Schorr,2011	6	12	6 (50%)	54,2±13.0	Home	5–6 times/week for≥ 6 hours	randomized trial	Y	Y	Y	7
Sikkes,2009	12	14	13 (92.9%)	47.0±7.8	Home	6 times/week for 8 hours	prospective non-randomized	Y	N	Y	5
Spanner,2003	18	13	10 (76.9%)	44,2±6.4	Home	5–6 times/week for 6–8 hours	prospective controlled non-randomized	N	Y	Y	7

Abbreviations: NHD: nocturnal hemodialysis.

Three studies excluded patients that had severe co-morbid conditions (for example, patients with diabetes mellitus, documented cancer, dementia, cardiac angina during the two week period prior to the study, active infections) and other relevant conditions such as secondary hyperparathyroidism with need of calcium-sensitizing therapy, central venous catheter, required erythrocyte transfusion, severe arterial hypertension, technical reasons, having pacemakers, or amputation of a major limb [33,37,38]. In another study, an average creatinine and urea clearance >10 ml/min/1.73m^2^ were exclusion criteria [17]. In certain studies, short life expectancy [39], inappropriate housing and inability to speak English [40], lacking the physical ability or cognitive function to train for NHD [41], as well as the absence of a partner to assist with in-home NHD [14] were reasons for exclusion. Five studies described no specific exclusion criteria [18,34–36,42]. Major inclusion criteria in all studies were the receptiveness to participate and the capability to be trained for NHD.

Inclusively, in all 13 studies, 282 patients made the transition from CHD to NHD. Four of these 13 studies (comprising 168 patients on NHD) had a control group of CHD patients (208 patients on CHD) [17,37,41,42]. The other nine studies (comprising 114 NHD patients) compared their outcomes to measurements at baseline prior to patients beginning NHD [14,18,33–36,38–40]. The length of the intervention period on NHD ranged between eight weeks and three years.

The score on the Newcastle Ottawa Scale for cohort studies ranged from 3 to 8, one study was considered poor [33], seven were considered fair [14,18,34,35,38–40], and five were considered good [17,36,37,41,42]. There were no randomized controlled trials.

### Biochemical nutritional measurements

#### Control group controlled studies

**Fig 2:**
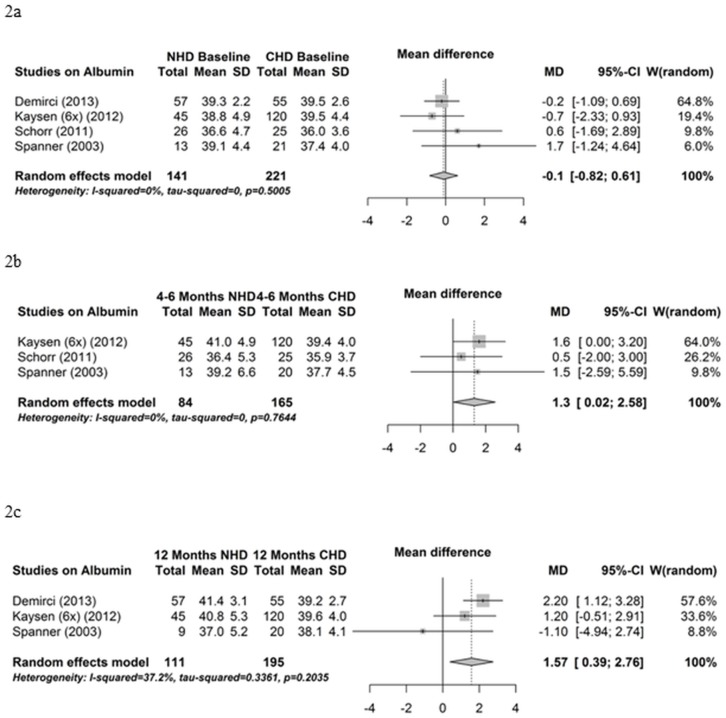
Albumin—control group controlled studies. (2a) Forest plot comparing albumin in NHD patients at baseline (before transition from CHD to NHD) and control CHD patients. (2b) Forest plot comparing albumin in NHD patients after 4–6 months on NHD and control CHD patients after 4–6 months follow up. (2c) Forest plot comparing albumin in NHD patients after 12 months on NHD and control CHD patients after 12 months follow up.

Serum albumin was measured in 12 studies. A meta-analysis comparing NHD patients and control patients that remained on CHD is performed on the results from four studies. A meta-analysis for the comparison of albumin levels at baseline (before the transition from CHD to NHD) between NHD patients and CHD patients demonstrated no differences (mean difference -0.1 g/l, 95% CI -0.82; 0.61, P = 0.78) (Fig 2a). Influence analyses and funnel plot indicated no bias (data not shown). The meta-analysis of the comparison of the means of three studies that compared NHD patients with a follow up period of 4–6 months with control patients that continued on CHD for 4–6 months showed a borderline significant increase in serum albumin in NHD patients (mean difference 1.3 g/l, 95% CI 0.02; 2.58, P = 0.05) (Fig 2b). The influence analysis shows a modification in the results after removing one particular study (Table 3). The funnel plot indicated no bias (data not shown). The meta-analysis comparing NHD patients at 12 months following the transition compared with control patients after 12 months of follow-up also indicated a significant increase in serum albumin (mean difference 1.57 g/l, 95% CI 0.39; 2.76, P = 0.01) (Fig 2c). The influence analysis showed a change in the results after omitting a particular study (Table 4). The funnel plot indicated no bias (data not shown).

**Table 3:** Influence analysis of albumin at 4–6 months of NHD versus controls on CHD.

	Mean difference	95% confidence interval	P-value
Omitting Kaysen (6x per week) (2012)	0.77	-1.36–2.91	0.479
Omitting Schorr (2011)	1.59	0.10–3.08	0.037
Omitting Spanner (2003)	1.28	-0.07–2.63	0.063
Pooled estimate	1.30	0.02–2.58	0.046

**Table 4:** Influence analysis of albumin at 12 months of NHD versus controls CHD.

	Mean difference	95% confidence interval	P-value
Omitting Demirci (2013)	0.72	-1.11–2.55	0.44
Omitting Kaysen (6x per week) (2012)	1.09	-1.97–4.14	0.486
Omitting Spanner (2003)	1.92	1.01–2.83	0.000
Pooled estimate	1.57	0.39–2.76	0.009

Identical results were obtained when the meta-analysis comparing albumin levels between NHD and CHD control patients was performed with the NHD group that dialyzed three times per week instead of the NHD group that dialyzed six times per week NHD (S1A, S1B and S1C Fig).

#### Baseline controlled studies

**Fig 3:**
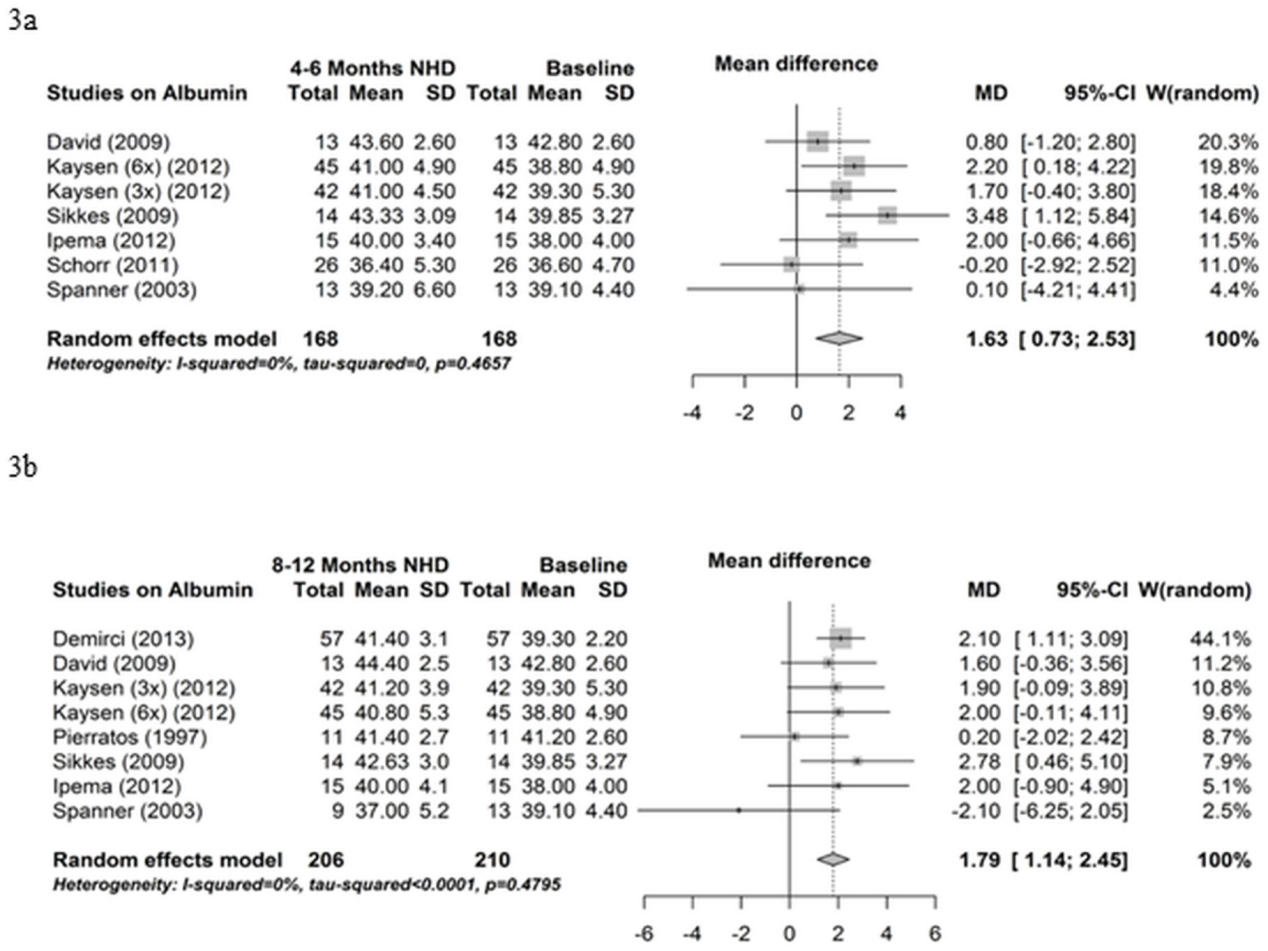
Albumin—baseline controlled studies. (3a) Forest plot comparing albumin in NHD patients at baseline (before beginning NHD) and after 4–6 months on NHD. (3b) Forest plot comparing albumin in NHD patients at baseline (before beginning NHD) and after 8–12 months on NHD.

The results of eight baseline controlled studies on albumin are exploited in a meta-analysis. Serum albumin significantly increased from baseline to 4–6 months NHD (mean difference 1.63 g/l, 95% CI 0.73; 2.53, P<0.001) (Fig 3a) and from baseline to 8–12 months NHD (mean difference 1.79 g/l, 95% CI 1.14; 2.45, P<0.001) (Fig 3b). Influence analysis and funnel plots indicated no bias (data not shown).

**Fig 4:**
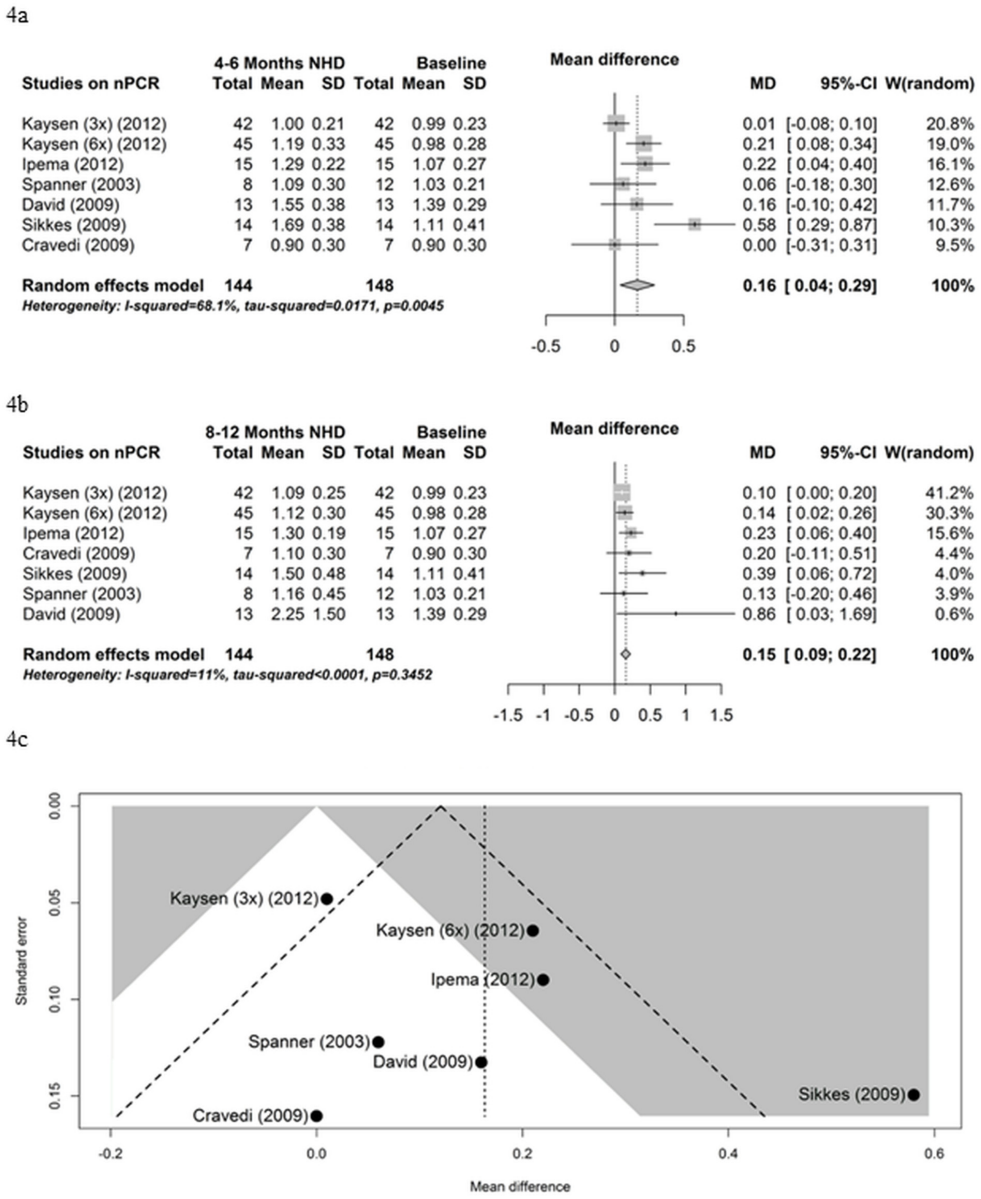
normalized Protein Catabolic Rate. (4a) Forest plot comparing nPCR in NHD patients at baseline (prior to beginning NHD) and after 4–6 months on NHD. (4b) Forest plot comparing nPCR in NHD patients at baseline (prior to beginning NHD) and after 8–12 months on NHD. (4c) Funnel plot comparing nPCR in NHD patients at baseline (prior to beginning NHD) and after 4–6 months on NHD.

Nine studies reported results on nPCR whereby six could be utilized in the meta-analysis. As illustrated in Fig 4a, nPCR significantly improved from baseline to 4–6 months on NHD (mean difference 0.16 g/kg/day, 95% CI 0.04; 0.29, P = 0.01). The homogeneity of effects was rejected in this meta-analysis by a p-value of 0.005. A test for funnel plot asymmetry revealed that two studies fell outside the broken lines of the funnel plot in the comparison of nPCR between baseline and 4–6 months [14,17] (Fig 4c). The influence analysis showed no changes after the omission of any single study. The nPCR also significantly improved from baseline to 8–12 months NHD (mean difference 0.15 g/kg/day, 95% CI 0.09; 0.22, P<0.001) (Fig 4b). In this analysis, the homogeneity of the effects was not rejected (P = 0.35). There was neither an outlying study from the funnel plot nor an influential study after omitting a single study.

CRP was measured in three studies [36–38]. Meta-analysis was not performed since only two of these studies were appropriate for this analysis. In two studies, CRP did not significantly differ between baseline and 12 months on NHD [36,38] whereas, in another study, CRP decreased significantly from baseline to 12 months on NHD [37]. In the latter study, the course of CRP values did not differ between the NHD group and the control group over the course of one year [37].

### Body composition

**Fig 5:**
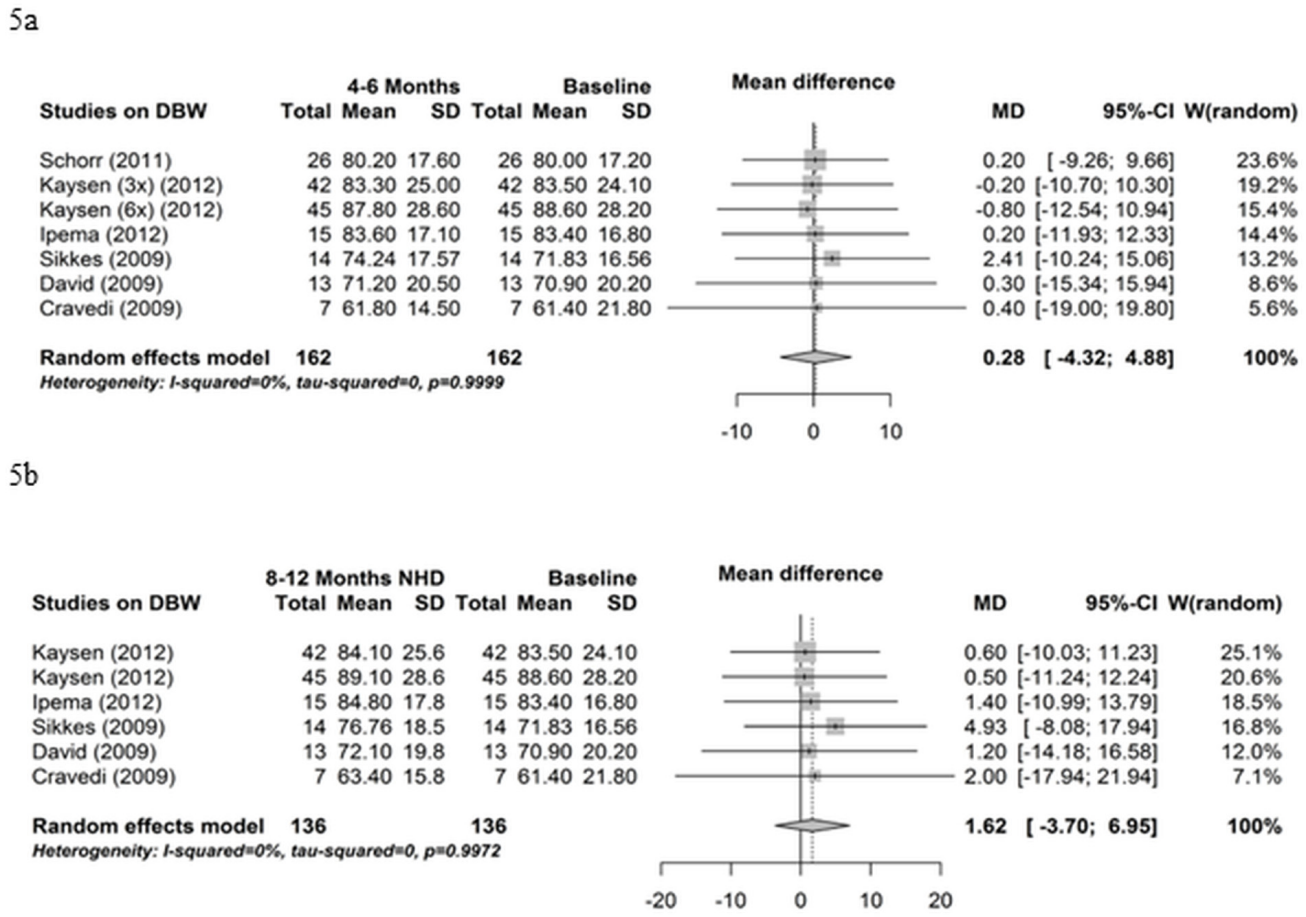
Dry body weight. (5a) Forest plot comparing dry body weight (DBW) in NHD patients at baseline (prior to beginning NHD) and after 4–6 months on NHD. (5b) Forest plot comparing dry body weight (DBW) in NHD patients at baseline (prior to beginning NHD) and after 8–12 months on NHD.

All thirteen studies reported data on the course of body weight. Six of these studies could be employed for the meta-analysis (Fig 5). Dry (post-dialysis) body weight did not change from baseline to 4–6 months NHD (mean difference 0.28 kg, 95% CI -4.32; 4.88, P = 0.9) (Fig 5a) and from baseline to 8–12 months NHD (mean difference 1.62 kg, 95% CI -3.70; 6.95, P = 0.55) (Fig 5b). Influence analyses and funnel plot indicated no bias (data not shown).

**Fig 6:**
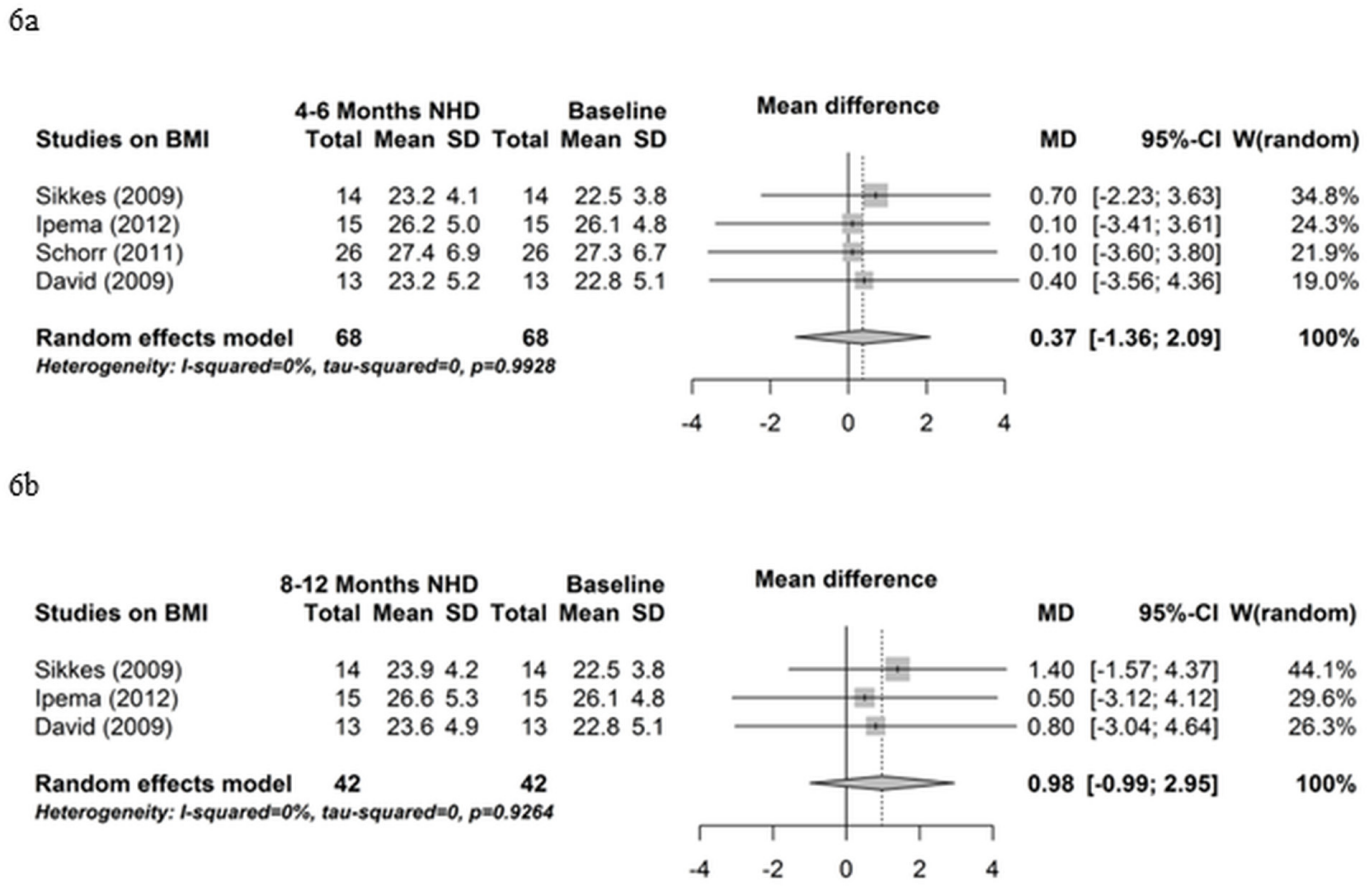
Body mass index. (6a) Forest plot comparing body mass index (BMI) in NHD patients at baseline (prior to beginning NHD) and after 4–6 months on NHD. (6b) Forest plot comparing body mass index (BMI) in NHD patients at baseline (prior to beginning NHD) and after 8–12 months on NHD.

Five studies reported data on BMI. As depicted in Fig 6, BMI did not change from baseline to 4–6 months (mean difference 0.37, 95% CI -1.36; 2.09, P = 0.68) (Fig 6a) and from baseline to 8–12 months (mean difference 0.98, 95% CI -0.99; 2.95, P = 0.33) (Fig 6b). Influence analyses and funnel plot indicated no bias (data not shown).

Lean body mass measured with bioimpedance analysis (BIA) was reported in two studies. One indicated a significant increase in lean body mass compared with the CHD control group after 12 months [37]. The other showed no significant change in lean body mass during an observation period of 12 months [17].

ECW, ICW, ECM, and BCM were measured with BIA in three studies. One study reported significant decreases in ECW normalized for weight and in ECW normalized for height after 12 months of follow-up compared with the control group [37]. Another study documented that the ratio of the ECM and BCM (ECM/BCM) decreased significantly from baseline to 12 months on NHD [38]. The third and largest study reported no significant changes in ECW, ICW, and total body water in NHD patients during 12 months of follow-up [17]. Meta-analysis was not performed because these studies used different outcome parameters.

Interdialytic weight was reported in two studies. One showed a significant increase from baseline to 8 months NHD [39] while the other indicated no change between baseline and 12 months of NHD [36].

Three studies measured fat mass. One study denoted a significant increase after 12 months on NHD compared with the control group [37]. Another study reported no significant change in the percentage of body fat during one year of NHD [42]. The third study ascertained no change in fat mass during 12 months on NHD compared with a control group [17]. The use of a different measurement of fat mass precluded a meta-analysis.

Two studies reported the upper arm muscle area whereby one showed no change in MUAMC from baseline to 8 months NHD [39]. The other study showed no change in arm muscle area from baseline to 18 months NHD [42].

**Fig 7:**
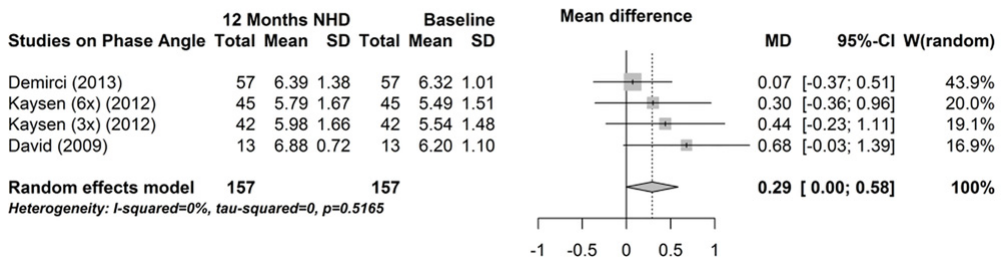
Forest plot comparing phase angle in NHD patients at baseline (prior to beginning NHD) and after 12 months on NHD.

Three studies reported phase angle and had divergent outcomes [17,37,38]. As illustrated in Fig 7, phase angle did not significantly change from baseline to 12 months (mean difference 0.29°, 95% CI -0.004; 0.584, P = 0.05), although there was a positive trend. Influence analysis and funnel plot indicated no bias (data not shown).

### Food records

**Fig 8:**
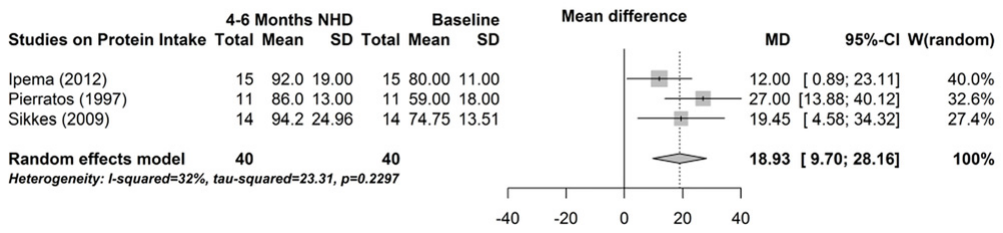
Forest plot comparing protein intake in NHD patients at baseline (prior to beginning NHD) and after 4–6 months on NHD.

Protein intake was measured in six studies, of which three could be used for the meta-analysis [14,39,40]. As shown in Fig 8, protein intake increased significantly from baseline to 4–6 months NHD (mean difference 18.9 g, 95% CI 9.7; 28.2, P<0.001). Influence analysis and funnel plot indicated no bias (data not shown).

**Fig 9:**
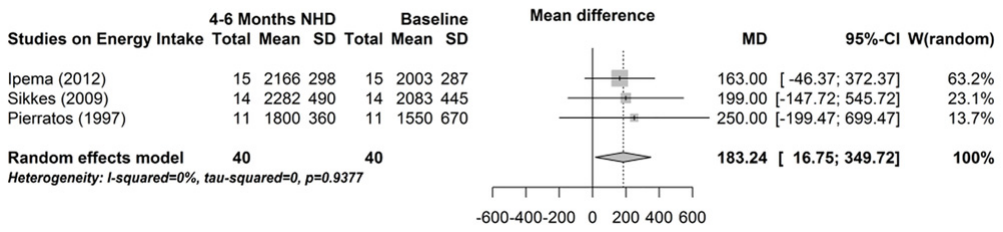
Forest plot comparing energy intake in NHD patients at baseline (prior to beginning NHD) and after 4–6 months on NHD.

Six studies reported energy intake, of which three could be used for meta-analysis [14,39,40]. As depicted in Fig 9, energy intake increased significantly between baseline and 4–6 months NHD (mean difference 183.2 kcal, 95% CI 16.8; 349.7, P = 0.03). The influence analysis showed a change in the results after omitting one particular study (Table 5). The funnel plot indicated no bias (data not shown). There is a meta-effect, however, the degree of evidence is not very convincing.

**Table 5:** Influence analysis of energy intake between baseline and after 4–6 months of NHD.

	Mean difference	95% confidence interval	P-value
Omitting Ipema (2012)	218	-56.5–492.6	0.120
Omitting Pierratos (1997)	173	-6.6–351.8	0.059
Omitting Sikkes (2009)	179	-11.3–368.3	0.065
Pooled estimate	183	16.8–349.7	0.031

Three studies measured carbohydrate intake and fat intake [14,39,41]. Meta analysis was not performed since only two of these studies were suitable [14,39]. None of the studies reported a significant change between baseline and 4 to12 months following the initiation of NHD [14,39,41].

## Discussion

In our meta-analyses, we ascertained that NHD is associated with significantly higher protein intake, nPCR, and serum albumin compared with CHD. The results for serum albumin are strengthened by the meta-analysis of studies that prospectively compared patients receiving NHD and those who remained on CHD, which showed similar positive effects. Energy intake also increased but with moderate evidence. In contrast, the body composition parameters of dry body weight, BMI, and phase angle did not change after the initiation of NHD.

There is no ‘gold standard’ for the assessment of nutritional status in hemodialysis patients. In clinical practice, a combination of parameters is utilized such as the Subjective Global Assessment, anthropometry (dry body weight, body composition), nPCR, and serum albumin [43]. This meta-analysis shows that serum albumin levels increased significantly following the transition from CHD to NHD. Serum albumin is a measure of visceral protein stores and has prognostic significance in hemodialysis patients [44,45]. Although the increase in serum albumin during NHD may reflect genuine improvement in nutritional status, non-nutritional factors may contribute to the rise in serum albumin, e.g., improvement of systemic inflammation and/or reduction of proteinuria as a result of loss of residual renal function. It is unknown whether the increase in serum albumin after the transition to NHD translates into lower mortality. Notably, serum albumin is also a negative acute phase protein. However, CRP values did not significantly change post initiation of NHD [36–38]. This suggests that the increase in serum albumin levels during NHD is related to higher protein intake. This is substantiated by our meta-analyses demonstrating that both protein intake and nPCR increased significant after the transition from CHD to NHD. These effects were still evident after 8–12 months of receiving NHD. In accordance with these findings is the positive meta-effect for energy intake. These findings strongly posit that patients consume more nutrition following the transition to NHD. Sikkes et al indeed ascertained that 50% of NHD patients had an increased appetite after one year on NHD and that appetite appeared to improve more in patients with a lower baseline nPCR [14].

Previous studies showed better survival rates in HD patients with increasing weight and BMI [46,47] whereas weight loss is most commonly associated with a deterioration of nutritional status [43]. In our meta-analysis, dry body weight and BMI did not significantly vary during NHD. However, a change in body weight is not a sensitive parameter of nutritional status since it is affected by changes in lean body mass and hydration status. Various authors suggested that more frequent and/or longer hemodialysis sessions are associated with better control of extracellular volume leading to lower blood pressure with less antihypertensive medication [12,48–50]. Therefore, increases in lean body mass during NHD may be disregarded as a result of a contemporary reduction in extracellular fluid. Only three studies measured changes in fluid status during NHD [17,37,38]. Demirci et al indeed reported a significant decrease in ECW normalized for height and in ECW normalized for weight while both parameters increased in the control group of CHD patients [37]. David et al also reported a significant decrease in ECM/BCM from baseline to 6 months while receiving NHD [38]. In contrast, body water parameters, including ECW, did not alter during NHD in the study of Kaysen et al [17]. The minimal number of studies and the divergent results preclude a firm conclusion with regard to a possible substitution of extracellular fluid by lean body mass during NHD.

Lean body mass was measured with BIA in two studies and yielded different outcomes [17,37]. Kaysen et al determined no differences in lean body mass between the three times per week NHD group and the six times per week NHD group, although an insignificant decreasing trend after four months was indicated [17]. The study of Demirci et al showed significant increases in lean body mass and in fat mass in the NHD patients compared with the CHD controls in the course of one year [37]. Bioimpedance phase angle is a novel technique that may be exploited as a predictor for impaired muscle function and as an indicator for nutritional status, health related quality of life, forthcoming hospitalizations, and mortality [51]. Phase angle was measured in three studies and an insignificant positive trend in NHD patients was found in the meta-analysis.

In two studies, the upper arm muscle area was measured [39,42]. Spanner et al. discovered a steady decline in arm muscle area throughout the study period in NHD patients that could be due to accompanying loss in body weight that was also evident [42]. No change in muscle arm circumference in NHD patients was found in a study by Ipema et al [39]. The upper arm muscle area can be used for an estimation of the lean body mass and can be a predictor of mortality in hemodialysis patients [52,53]. On the other hand, it is not a very sensitive measurement being limited in reproducibility and has a relatively large inter-observer variability [53]. Overall, it can be concluded that our analysis does not provide evidence that body composition changes during NHD.

Remarkably, patients receiving NHD had a significantly higher protein and energy intake but did not exhibit an increase in BMI or dry body weight. This counter-intuitive finding may be explained by a negative protein balance induced by the NHD treatment. Various studies showed that hemodialysis while fasting is associated with a negative protein balance [54–56]. During NHD, patients are asleep and generally do not eat. It is feasible that the significant increase in protein intake in NHD patients is offset by increased losses of amino acids and increased hemodialysis-associated catabolism. Alternatively, the failure of patients to increase physical activity in order to increase fat free mass may play a role in the negative protein balance. None of the included studies measured daily activity and exercise in NHD patients. Chan et al studied exercise duration and capacity in patients after conversion to NHD and found an improvement [57]. However, in a more recent study, it was found that NHD had no effect on physical performance, health, and functioning in the NHD group compared with the CHD group [58].

Although our systematic review and meta-analysis provides new information on the potential benefits of NHD, the results must be interpreted within the context of methodological limitations. First, most studies were constrained by a minimal sample size, the withdrawal of patients (on NHD) is not always described, a number of studies had incomplete dietary records for the participants, and, in several studies, the follow-up duration is relatively brief. Another limitation is the assumption that the experimental and control groups are independent. In cases with repeated measurement in the same group of subjects, such an assumption is not valid. However, meta-analysis programs do not account for the possibility of repeated measurements because these are rarely reported in complete detail. In these cases, however, our approach based on independence can be expected to underestimate the actual meta-effect. Considering the fact that there were only thirteen studies considered eligible for this review, we have decided not to exclude due to study design. This resulted in an extensively varied study design. Formal blinding of participants is impossible. To anticipate the limitations, several quality measurements were assessed. In all meta-analysis, we assessed homogeneity of effects, performed an influence analysis, and tested for funnel plot asymmetry. With a few exceptions, these tests did not provide evidence for bias. Notably, five out of the 14 meta-analyses were performed with only three studies. In three of these five meta-analyses a deviating influence analysis was found. Two of these were the meta-analyses of ‘control group controlled’ studies comparing albumin levels at 4–6 and at 12 months on NHD, respectively. The outcomes of these meta-analysis were used to corroborate the results of ‘baseline controlled studies’ comparing baseline with 4–6 and 8–12 months on NHD, respectively. The third meta-analysis with deviating influence analysis was on energy intake. Although, the three studies in this meta-analysis showed a uniform pattern, the deviating influence analysis precludes a definite conclusion on the effect of NHD on energy intake. There are no reviews published in regard to nutritional status in nocturnal hemodialysis patients to compare our results with or to be able to reconsider any type of disagreements in outcomes.

## Conclusions and Recommendations

This systematic review illustrates that NHD is associated with a significantly higher protein- and energy intake and increases in serum albumin and nPCR. Prospective randomized trials should be conducted to add more evidence to the effect of the transition from CHD to NHD on nutritional status in its various facets: food intake, laboratory parameters, and body composition. Additionally, future studies should address the important question whether the higher protein intake during NHD translates into a lower morbidity and mortality. Finally, more research should be devoted to the effect of combined treatment with intensive NHD and an exercise program on body composition.

## Supporting Information

S1 Fig Albumin (Kaysen 3x per week NHD instead of 6x per week NHD [17]). **(A)** Forrest plot comparing albumin in NHD patients on baseline (before transition from CHD to NHD) and control CHD patients. **(B)**Forrest plot comparing albumin in NHD patients after 4–6 months on NHD and control CHD patients after 4–6 months follow up. **(C)** Forrest plot comparing albumin in NHD patients after 12 months on NHD and control CHD patients after 12 months follow up.
(PDF)

S1 File Protocol systematic review. (PDF)

S1 Table PRISMA 2009 checklist. Checklist of this systematic review and meta-analyses.
(PDF)

S2 Table Summary of the results of the 13 studies. Summary of the results of the 13 studies on the nutritional status of nocturnal hemodialysis patients.
(PDF)
