# Supplementary figures and images for: Nutritional Status in Nocturnal Hemodialysis Patients – A Systematic Review with Meta-Analysis
Source: PLoS One. 2016 Jun 20;11(6):e0157621. doi: 10.1371/journal.pone.0157621 (PMC4913934; doi:10.1371/journal.pone.0157621)

S1 Figs.

S1a Fig.

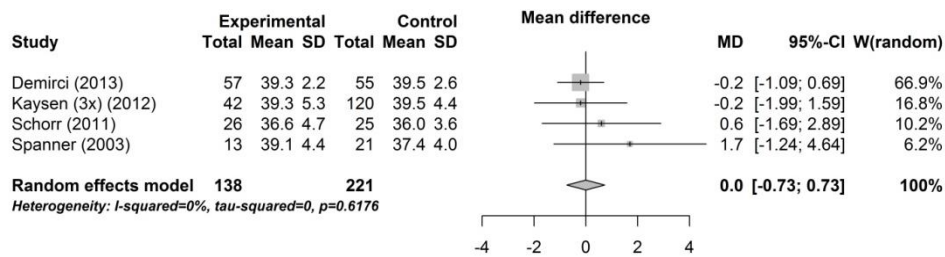

S1b Fig.

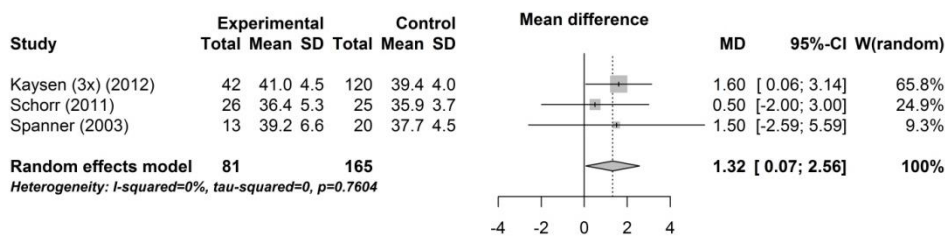

S1c Fig.

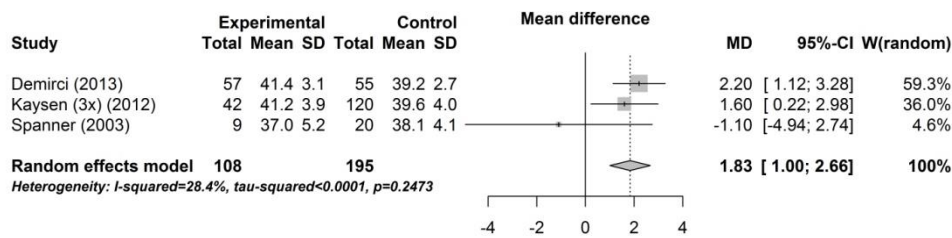

Supplement: S1 Fig — (A) Forrest plot comparing albumin in NHD patients on baseline (before transition from CHD to NHD) and control CHD patients. (B)Forrest plot comparing albumin in NHD patients after 4–6 months on NHD and control CHD patients after 4–6 months follow up. (C) Forrest plot comparing albumin in NHD patients after 12 months on NHD and control CHD patients after 12 months follow up. (PDF) [file pone.0157621.s001.pdf]
