# Supplementary material for: Nutritional Status in Nocturnal Hemodialysis Patients – A Systematic Review with Meta-Analysis
Source: PLoS One. 2016 Jun 20;11(6):e0157621. doi: 10.1371/journal.pone.0157621 (PMC4913934; doi:10.1371/journal.pone.0157621)
Supplement: S1 File — (PDF) [file pone.0157621.s002.pdf]

S1 File. Protocol

A systematic review of the effect of nocturnal hemodialysis on nutritional status and food intake.

Aim of the review

The aim of this study is to review the effects of the transition from conventional hemodialysis to nocturnal hemodialysis on nutritional status (laboratory parameters, body composition, and food intake).

Objectives

To assess the effects of the transition from conventional hemodialysis to nocturnal hemodialysis in adult (≥18 years) dialysis patients. This review will include studies that measured:

the effect of the transition from conventional hemodialysis to nocturnal hemodialysis on the nutritional status and body composition (nPCR, Kt/V, albumin, CRP, BMI, fat mass, fat free mass, lean mass, upper arm muscle circumference);

the effect of the transition from conventional hemodialysis to nocturnal hemodialysis on food intake, as measured by energy intake, macronutrients like protein, carbohydrates, and fat, micronutrients like sodium, potassium, calcium, and phosphate.

Inclusion and exclusion criteria

To be included in the review, studies must address one or more of the following factors: nocturnal hemodialysis/haemodialysis, nutritional state/ status, and/or food intake (in title or abstract), relation or overlap with: malnutrition, protein intake, food, frequent nocturnal hemodialysis, night.

The following terms for are also considered for inclusion:

- malnutrition
- protein wasting
- energy wasting

- food intake
- frequent hemodialysis/haemodialysis
- daily hemodialysis/ haemodialysis
- The participants in the studies must be adult hemodialysis patients ($\geq$18 years)

Types of studies

- randomized controlled trials, controlled trials, cohort studies
- observational studies with a comparison group, case-control
- prospective longitudinal before and after studies

systematic reviews with meta-analyses (when relevant systematic reviews are found, these will be summarized and the results will be completed with more recent primary research).

Methods

Search strategy

Electronic searching; The following databases will be searched:

- MEDLINE / Pubmed
- EMBASE
- CINAHL
- The Cochrane Library

Other additional search strategies will include:

- Examination of reference lists from relevant studies
- Citation searching

Search strategies will be documented in detail.

Studies published between January 1st, 1990 (first publication 1996?) through February 1st, 2014, written in English and published in a peer-reviewed journal, containing primary research were considered for the review.

Screening and identification

<u>Screening of the titles:</u> The titles of potential relevant papers will be screened against the inclusion and exclusion criteria by the two researchers and one supervisor (K. Ipema).

When in doubt based on title, the abstract will be opened and reviewed. Titles and papers will be send to Refworks.

<u>Screening of the abstracts:</u> The abstracts of the papers send to Refworks in the first round will be screened against the inclusion and exclusion criteria. Abstracts that do not meet the inclusion criteria will be removed from the Refworks file, but stored in another 'exclusion' file.

<u>Eligibility:</u> Full-texts articles will be assessed for eligibility. The two researchers will check the eligibility of articles individually and discuss disagreements. The supervisor will give a second opinion. Disagreements will be solved by discussion between the researchers and the supervisor.

The two researchers will review the papers and accurately check them against the inclusion criteria. Any uncertainty will be discussed with the supervisor.

Full papers will be screened for the following inclusion/exclusion criteria:

- Study design (the design of the study was described in sufficient detail that could be replicated)
- Population (sample size, randomized Y/N, characteristics: age, dialysis vintage, gender, previous transplantation)
- NHD and control groups
- Dialysis regimen (number of sessions per week, duration of dialysis sessions, blood flow, dialysate flow, type of dialyser)
- Nutritional state outcomes
- Body composition outcomes
- Data analysis

Results will be incorporated in the PRISMA flow chart.

Included (Results)

Data extraction and analysis

From the included papers, details on patients characteristics, research groups, laboratory measurements, body composition outcomes, food intake outcomes, and quality will be extracted and compiled in a standard format by the researchers. The supervisor will independently check the data extraction forms for accuracy and detail, disagreements will be resolved by consensus or another reviewer.

Assessment of risk of bias in included studies

The methods used for assessing risk of bias of individual studies (including specification of whether this was done at the study on outcome level), and how this information is to be used in any data synthesis will be described.

Assessment of risk of bias across studies

Any assessment of risk of bias that may affect the cumulative evidence will be specified (such as publication bias, selective reporting within studies).
