# Supplementary material for: Nutritional Status in Nocturnal Hemodialysis Patients – A Systematic Review with Meta-Analysis
Source: PLoS One. 2016 Jun 20;11(6):e0157621. doi: 10.1371/journal.pone.0157621 (PMC4913934; doi:10.1371/journal.pone.0157621)
Supplement: S2 Table — Summary of the results of the 13 studies on the nutritional status of nocturnal hemodialysis patients. (PDF) [file pone.0157621.s004.pdf]

S2 Table. Summary of the results of the 13 studies on the nutritional status of nocturnal hemodialysis patients.

Author	Results of biochemical measurements on nutrition	Results of body composition	Results of food records	Conclusion of the authors	Comments
Alloatti, 2002	**PCR**: g/day, CHD baseline 1.12 (0.30), after ≥6 months NHD 1.20 (0.33)<br>**Serum albumin**: g/L, CHD baseline 38.5 (6.0), after ≥6 months NHD 39.8 (6.0)	**Post-dialysis weight**: kg, CHD baseline 68.5 (9.6), after ≥6 months NHD 70.8 (10.7); P<0.01		Good results of: toxin removal, water balance regulation, and maintenance of an adequate nutritional status.	Second measurement is after 6 months NHD, not clear whether it is 6 or 36 months.
Cravedi, 2009	**nPCR:** g/kg/day CHD, baseline 0.9 (0.3), 6 months 0.9 (0.3), 12 months 1.0 (0.4), NHD, 18 months 0.9 (0.3), 24 months 1.1 (0.3), 30 months 1.1 (0.3), 36 months 1.1 (0.4)	**Post-dialysis body weigh**t: kg, CHD baseline (at 12 months) 61,4 (21.8), NHD 6 months (at 18 months) 61.8 (14.5), NHD 12 months (at 24 months) 63.4 (15.8), p<0.001 (measured from Fig. 1.B of the original article)		Improvement of nutritional status.	Results of body weight were shown in a figure. Only. Results were measured manually from the figure..
David, 2009	**Albumin:** g/L, baseline 42.8 (2.6), 3 months 43.3 (1.6), 6 months 43.6 (2.6), 9 months 43.2 (3.1), 12 months 44.4 (2.5), ns<br>**nPCR:** g/kg/day, baseline 1.39 (0.29), 3 months 1.71 (0.23) [1], 6 months 1.55 (0.38), 9 months 1.78 (0.37) [1], 12 months 2.25 (1.5), ns<br>**CRP:** mg/L, baseline 10.2 (13.8), 3 months 7.1 (9.8), 6 months 4.4 (3.6), 9 months 4.0 (4.2), 12 months 3.4 (4.1), ns.	**Dry body weight:** kg, baseline 70.9 (20.2), 3 months 71.0 (21), 6 months 71.2 (20.5), 9 months 71.6 (19.5) [1], 12 months 72.1 (19.8) [1] P= 0.04.<br>**BMI:** kg/m^2, baseline 22.8 (5.1), 3 months 22.9 (5.1), 6 months 23.2 (5.2), 9 months 23.4 (5.0) [1], 12 months 23.6 (4.9) [1] P= 0.009.<br>**Phase angle:** Degrees, baseline 6.2 (1.1), 6 months 6.91 (0.73) [1], 12 months 6.88 (0.72) [2], P=0.001<br>**ECM/BCM:** Mean (± SD) baseline 0.91 (0.16), 6 months 0.78 (0.09) [1], 12 months 0.78 (0.1) [1] P= 0.002		Improvement in nutritional control.	

Demirci, 2013	**Serum albumin:** g/dL, NHD baseline 3.93 (0.22), after 12 months 4.14 (0.31) change 0.19 (0.21), CHD baseline 3.95 (0.26), after 12 months 3.92 (0.27), change -0.02 (0.2), between group comparison (95% CI) 0.2 (0.1; 0.29), Δ [3] (95% CI) 0.19 (0.1; 0.29) P= <0.001<br>**CRP:** mg/dl, NHD baseline 1.67 (2.01), 12 months 1.32 (1.27), change -0.34 (1.26), CHD mg/dl, mean (± SD) baseline 1.53 (1.46), 12 months 1.65 (1.61), change 0.1 (1.6) Between group comparison (95% CI) baseline 0.14 (-0.54; 0.82), 12 months -0.33 (-0.88; 0.18), change 0.45 (-1.03; 0.1) Δ (95% CI) -0.35 (0.81; 0.1) P= 0.12	**Dry lean mass:** Change in kg, NHD baseline 12.2 (4.1), after 12 months 12.7 (4.1), change 0.5 (1.2), CHD baseline 11.5 (3.9), after 12 months 11.4 (3.9), change -0.1 (0.9), between group comparison (95% CI) 0.5 (0.1; 0.9) Δ [3] (95% CI) 0.58 (0.17; 0.9), P=0.006<br>**Fat mass:** Change in kg, NHD baseline 16.3 (8.7), after 12 months 17.4 (8.4), change 1.1 (2.5), CHD baseline 16.5 (7.7), after 12 months 16.1 (7.6), change -0.4 (3.2), between group comparison (95% CI) 1.5 (0.4; 2.7) Δ [3] (95% CI) 1.8 (0.8; 2.7), P<0.001<br>**Phase angle**: Change in phase angle degrees, NHD baseline 6.32 (1.01), after 12 months 6.39 (1.38), change 0.06 (1.05), CHD baseline 6.01 (1.13), after 12 months 5.71 (1.33), change -0.29 (0.9), between group comparison (95% CI) 0.35 (-0.02; 0.74) Δ [3] (95% CI) 0.37 (0.01; 0.7) P=0.04<br>**ECW/weight**: Change in l/kg, NHD baseline 0.26 (0.03), after 12 months 0.25 (0.03), change -0.01 (0.02), CHD baseline 0.26 (0.02), after 12 months 0.26 (0.01), change 0.01 (0.9), between group comparison (95% CI) -0.01 (-0.02; -0.009) Δ [3] (95% CI) -0.01 (-0.02; -0.01) P <0.001<br>**ECW/height**: Change in l/m, NHD baseline 10.22 (1.03), after 12 months 9.97 (1.12), change -0.25 (0.8), CHD baseline 10.21 (1.43), after 12 months 10.46 (1.33), change 0.25 (0.7), between group comparison (95% CI) -0.5 (-0.8; -0.22) Δ [3] (95% CI) -0.44 (-0.68; -0.20) P <0.001		In-centre NHD improves nutritional status and facilitates volume control.	
Ipema, 2012	**PCR:** g/day, baseline CHD 80 (27), 4 months NHHD 94 (22), 8 months NHHD 96 (20) P=0.001, Effect size (95% CI for difference with baseline CHD), P<0.05, 0-4 months 4.7; 24.7 g/day, 0-8 months 5.3; 27.0 g/day<br>**nPCR:** g/kg/day, baseline CHD 1.07 (0.27), 4 months NHHD 1.29 (0.22), 8 months NHHD 1.30 (0.19) P=0.001 [2] Effect size (95% CI for difference with baseline CHD),P<0.05, 0-4 months 0.04; 0.4 g/day, 0-8 months 0.4; 4.14 g/day<br>**Serum albumin:** g/L, baseline CHD 38 (4.0), 4 months NHHD 40 (3.4), 8 months NHHD 40 (4.1) P=0.232 [4]	**BMI:** kg/m^2, baseline CHD 26.1 (4.8), 4 months NHHD 26.2 (5.0), 8 months NHHD 26.6 (5.3) P=0.138 [4]<br>**Post-dialysis weight:** kg, baseline CHD 83.4 (16.8), 4 months NHHD 83.6 (17.1), 8 months NHHD 84.8 (17.8) P=0.183 [4]<br>**MUAMC:** cm, baseline CHD 27.0 (4.2), 4 months NHHD 27.4 (4.4), 8 months NHHD 27.4 (4.1) P=0.392 [4]<br>**Interdialytic weight:** Change in kg/24 hours, baseline CHD 1.2 (0.7), 4 months NHHD 1.5 (0.6), 8 months NHHD 1.7 (0.6) P=0.034 [5] Effect size (95% CI for difference with baseline CHD) [6] 0-4 months -0.3; 0.9 kg/24 hours, 0-8 months 0.02; 1.1 kg/24 hours, P<0.05	**Energy intake:** Kcal/day, baseline CHD 2003 (287), 4 months NHHD 2166 (298), 8 months NHHD 2183 (388) P=0.088 [4]<br>**Protein intake:** g/day, baseline CHD 80 (11), 4 months NHHD 92 (19), 8 months NHHD 89 (19) P=0.023 Effect size (95% CI for difference with baseline CHD) 0-4 months 0.2; 22.8 g/day, P<0.05, 0-8 months -4.3; 21.0 g/day<br>**Carbohydrate intake:** g/day, baseline CHD 236 (44), 4 months NHHD 237 (36), 8 months NHHD 252 (46) P=0.369 [4]<br>**Fat intake:** g/day, baseline CHD 82 (17), 4 months NHHD 91 (24), 8 months NHHD 91 (29) P=0.103 [4]	The transition from CHD to NHHD has a positive effect on nutritional intake, in particular, protein intake.	After the transition to NHD the first measurement was done after 4 months of NHD; the second measurement was done after 8 months NHD.
Kaysen, 2012	**Serum albumin:** Daily Trial 3xweek g/dL, baseline 3.95 (0.44), 4 months 3.94 (0.4), 12 months 3.96 (0.4) [6] Change from baseline 4 months -0.02 (0.03), 12 months 0.00 (0.03)Nocturnal Trial 3xweek g/dL, baseline	**Post-dialysis weight:** Daily Trial 3x week kg, baseline 78.9 (19.76), 4 months 79.11 (19.9), 12 months 79.19 (19.86) [6] Change from baseline 4 months 0.37 (0.28), 12 months 0.23 (0.45) Nocturnal Trial 3x week kg, baseline 83.45 (24.08), 4 months 83.3 (25.03), 12 months 84.05		Frequent nocturnal hemodialysis yielded no net effect on parameters of nutritional status or	For good comparison we only showed of the daily trial the 3 times weekly sessions and

	3.93 (0.53), 4 months 4.1 (0.45), 12 months 4.12 (0.39) [6] Change from baseline 4 months 0.17 (0.05), 12 months 0.20 (0.05) Nocturnal Trial 6xweek g/dL, baseline 3.88 (0.49), 4 months 4.1 (0.49), 12 months 4.08 (0.53) [6] Change from baseline 4 months 0.2 (0.05), 12 months 0.19 (0.05)Treatment comparison 6x vs. 3x, 4 months 0.03 (-0.10; 0.16) P=0.65, 12 months -0.01 (-0.14; 0.12), P=0.88<br>**ePCR:** Daily Trial 3xweek g/day, baseline 64.67 (17.86), 4 months 65.09 (19.09), 12 months 64.26 (20.02) [6] Change from baseline 4 months 0.03 (1.19), 12 months -0.35 (1.29)Nocturnal Trial 3xweek g/day, mean (± SD) baseline 62.42 (21.6), 4 months 63.28 (21.49), 12 months 69.97 (24.23) [6] Change from baseline 4 months 1.65 (3.24), 12 months 6.3 (3.33) Nocturnal Trial 6x week g/day, baseline 62.86 (21.15), 4 months 70.96 (22.05), 12 months 74.55 (38.81) [6] Change from baseline 4 months 7.41 (3.24), 12 months 11.94 (3.45)Treatment comparison 6x vs. 3x, 4 months 5.76 (-2.42; 13.94) P=0.17, 12 months 5.65 (-2.98; 14.27) P=0.20	(25.64) [6] Change from baseline 4 months -0.44 (0.45), 12 months 0.36 (0.79) Nocturnal Trial 6x week kg, baseline 88.55 (28.19), 4 months 87.8 (28.56), 12 months 89.07 (28.56) [6] Change from baseline 4 months -0.45 (0.44), 12 months 0.88 (0.78)Treatment comparison 6x vs. 3x, 4 months -0.02 (-1.25; 1.22) P=0.98, 12 months 0.51 (-1.66; 2.69) P=0.64<br>**Phase angle:** Daily Trial 3xweek degrees, baseline 5.21 (1.21), 4 months 5.32 (1.44), 12 months 5.34 (1.58) [6] Change from baseline 4 months 0.01 (0.13), 12 months -0.02 (0.15)Nocturnal Trial 3x week degrees, baseline 5.54 (1.48), 4 months 5.76 (1.48), 12 months 5.98 (1.66) [6] Change from baseline 4 months 0.16 (0.24), 12 months 0.37 (0.22) Nocturnal Trial 6x week degrees, baseline 5.49 (1.51), 4 months 5.81 (1.69), 12 months 5.79 (1.67) [6] Change from baseline 4 months 0.25 (0.24), 12 months 0.32 (0.23). Treatment comparison 6x vs. 3x, 4 months 0.09 (-0.53; 0.72) P= 0.77, 12 months -0.05 (-0.66; 0.56) P=0.87<br>**Lean body mass:** Daily Trial 3xweek kg, baseline 44.0 (10.2), 4 months 44.2 (9.7), 12 months 45.0 (9.6) [6] Change from baseline 4 months 0.53 (0.26), 12 months 0.58 (0.32) Nocturnal Trial 3x week kg, baseline 46.3 (11.7), 4 months 46.1 (11.5), 12 months 44.8 (11.4) [6] Change from baseline 4 months -0.23 (0.41), 12 months -0.04 (0.61) Nocturnal Trial 6x week kg, baseline 47.4 (12.5), 4 months 47.2 (12.2), 12 months 48.2 (12.0) [6] Change from baseline 4 months -1.34 (0.41), 12 months -0.49 (0.63)Treatment comparison 6x vs. 3x, 4 months -1.11 (-2.25; 0.04) P= 0.057, 12 months -0.45 (-2.18; 1.28) P=0.61<br>**% Adiposity**: Daily Trial 3xweek kg, baseline 37.6 (13.7), 4 months 36.8 (13.4), 12 months 37.3 (12.8) [6] Change from baseline 4 months -0.23 (0.29), 12 months -0.09 (0.41) Nocturnal Trial 3x week kg, baseline 37.9 (14.6), 4 months 37.7 (14.5), 12 months 37.5 (15.1) [6] Change from baseline 4 months -0.53 (0.57), 12 months -0.17 (0.81) Nocturnal Trial 6x week kg, baseline 40.9 (17.7), 4 months 42.5 (18.1), 12 months 44.4 (18.5) [6] Change from baseline 4 months 0.25 (0.56), 12 months 1.73 (0.82) Treatment comparison 6x vs. 3x, 4 months 0.78 (-0.78; 2.35) P= 0.32, 12 months 1.90 (-0.36; 4.17) P=0.10<br>**TBW, ECW, ICW and BCM**: no significant changes in the NHD groups.		body composition.	not the daily HD of 6 times per week. The nPCR was not shown in the original article, but delivered after questioning for them.
Maduel, 2011l	**nPCR:** g/kg/day, all patients: baseline 1.24 (0.4), 3 months 1.40 (0.5), 6 months 1.44 (0.6), 9 months 1.44 (0.7), 12 months 1.36 (0.7)<br>**Albumin:** mg/dL all patients: baseline 3.98 (0.4), 3 months 3.93 (0.3), 6 months 3.92 (0.3), 9 months 3.88 (0.2), 12 months 3,90(0.3)	**Body weight:** kg, all patients: baseline 70.1 (19), 3 months 70.6 (19), 6 months 71.3 (19) (P<0.05), 9 months 71.7(19) (P<0.01), 12 months 72.2 (19) (P<0.01)<br>**Weight gain:** kg, all patients: baseline 2.62 (1.1), 3 months 3.24 (1.2), 6 months 2.95 (1.0), 9 months 3.17 (1.5), 12 months 3.33 (1.4)		Nocturnal every-other-day OL-HDF could be a good alternative since also improvement in nutritional status was	Not suitable for the meta-analysis because online HDF during NHD was used.

	**CRP:** mg/L, all patients: baseline 0.97 (1.6), 3 months 0.98 (1.3), 6 months 0.69 (0.8), 9 months 0.96 (1.4), 12 months 0.73 (1.0)			observed.	
McPhatter, 1999	**Serum albumin:** mg/dL, (mean, SD not available): 6 months before NHHD 3.4, 6 months after start NHHD 4.1	**Body weight:** Weight gain of 2% to 3% in the first year. No data shown.	**Energy intake:** Kcal/day, (mean, SD not available), baseline 1801 (1208; 2777), 3 months 2045 (1454; 2654), 6 months 2150 (1721; 3120)<br>**Protein intake:** g/day, (mean, SD not available), baseline 76 (45 to 107), 3 months 87 (56 to 107), 6 months 80 (56 to 113)	NHHD patients show healthy eating, adequate kilocalorie and protein intake, and maintaining dry weight and protein stores.	The results are not shown all in numbers, but in figures. The standard deviations are not given and in the figures it is not clear whether the standard deviation is used.
O'Sullivan, 1998	**Serum albumin:** g/dL, pre-study 3.63 (0.52), study period 3.68 (0.41), post-study 4.00 (0.26)<br>Pre-study vs. NHD P<0.10<br>Post-study vs. NHD P>0.10<br>**PCR**: g/kg/day, pre-study 1.07 (0.12), study period 1.27 (0.20), post-study 1.19 (0.26)<br>Pre-study vs. NHD P=0.075<br>Post-study vs. NHD P>0.10	**Body weight**: Not significant. No data shown.	**Energy intake:** Kcal/day,pre-study 2223.33 (1024.04) vs. study period 2628.33 (928.09) P=0.029	Higher doses of hemodialysis benefit nutrition, improve biochemical variables, and may improve many hormonal systems.	Very low amount of participants, 4 patients. The study time was only 8 weeks.
Pierratos, 1997	**Serum albumin:** g/L, pre-study CHD 41.2 (2.6), study period NHD 41.4 (2.7)	**Body weight:** Overall increase by 1.0±3.0 kg in 12 months (ns). No data shown.	**Protein intake**: within the first 6 months from 59 (18) to 86 (13) in g/day P=0.004, or 1.0 (0.3) to 1.44 (0.2) in g/kg/day P=0.009<br>**Energy intake:** Kcal/day, before conversion to NHD 1550 (670) to 1800 (360) at 6 m after conversion (ns)	An increase in the protein intake was found on nocturnal hemodialysis. Some of the patients gained significant weight (up to 5.5 kg).	The exact data of the body weight was not shown.
Schorr, 2011	*For subjects completing food diaries: (NHD n = 12, CHD n = 11)*<br>**Serum albumin:** g/L, NHD baseline 37.1 (4.4), 6 months 37.8 (4.0). CHD baseline 36.6 (3.5), 6 months 35.0 (3.5), change from baseline to exit: 0.7 (3.6) NHD (n=12), -1.6 (4.0) CHD (n=11), between group comparison (95% Cl): 2.3 (-0.97; 5.6)<br><br>*For all study subjects: n=51 (NHD n=26, CHD n=25)*<br>**Serum albumin:** g/L, NHD baseline 36.6 (4.7), 6 months 36.4 (5.3)<br>CHD baseline 36.0 (3.6), 6 months 35.9 (3.7), change from baseline to exit: -0.2 (4.5) on NHD, -0.2 (4.1) on CHD. Between group comparison (95% Cl): 0.0 (-2.4; 2.4)	*For subjects completing food diaries: (NHD n=12, CHD n=11)*<br><br>*For all study subjects: n=51 (NHD n=26, CHD n=25)*<br>**Weight:** kg, NHD baseline 80.0 (17.2), 6 months 80.2 (17.6), CHD baseline 69.3 (19.4), 6 months 69.0 (18.1), change from baseline to exit: 0.1 (3.3) on NHD, -0.3 (2.8) on CHD, between group comparison (95% Cl): 0.5 (-1.3; 2.2)<br>**BMI:** kg/m^2, NHD baseline 27.3 (6.7), 6 months 27.4 (6.9), CHD baseline 23.5 (5.5), 6 months 23.4 (5.0), change from baseline to exit: 0.05 (1.14) on NHD, -0.12 (0.96) on CHD, between group comparison (95% Cl): 0.17 (-0.43; 0.76)	*For subjects completing food diaries: (NHD n=12, CHD n=11)*<br>**Carbohydrate intake:** g/day, median (IQR) change from baseline to exit: -17.7 (-56.9; 53.1) on NHD, 9.7 (-56.9; 53.7) on CHD. Between group comparison (95% Cl) P=0.81<br>**Protein intake:** g/kg/day, median (IQR) change from baseline to exit: -0.07 (-0.13; 0.21) on NHD, 0.07 (-0.15; 0.44) on CHD. Between group comparison (95% Cl) P=0.67<br>**Fat intake:** g/day, median (IQR) change from baseline to exit: 4.3 (-23.7; 36.9) on NHD, -4.0 (-11.2; 17.6) on CHD. Between group comparison (95% Cl) ns.<br>*For all study subjects: n=51 (NHD n=26, CHD n=25)*	NHD subjects demonstrated a small increase in weight and BMI. Whether these dietary changes translate to improvements in nutritional status remains to be determined.	The results of the food records are not used in the meta analysis because the results are shown in median (IQR).

Sikkes, 2009	**nPCR:** g/kg, baseline 1.1 (0.1), 3 months 1.6 (0.1), 6 months 1.6 (0.1), 12 months 1.5 (0.1) P= 0.058 **Serum albumin:** g/L, baseline 40 (1), 3 months 43 (0), 6 months 43 (1), 12 months 42 (1) P= 0.001	**Body weight:** kg, baseline 71 (4), 3 months 74 (4), 6 months 74 (4), 12 months 76 (5) P=0.001 **BMI:** kg/m^2, baseline 22 (1), 3 months 23 (1), 6 months 23 (1), 12 months 24 (1) P=0.001	**Energy intake:** kcal/day, baseline 2083 (119), 3 months 2305 (132), 6 months 2282 (131), 12 months 2213 (159) P=0.220 **Protein intake: g/kg**, baseline 1.1 (0.1), 3 months 1.3 (0.1), 6 months 1.3 (0.1), 12 months 1.3 (0.1) P= 0.024 **Protein intake in g/day,** baseline 74 (4), 3 months 90 (6), 6 months 93 (6), 12 months 92 (6) P=0.003 **Carbohydrate intake:** g/day, baseline 263 (17), 3 months 287 (20), 6 months 262 (18), 12 months 249 (18) P=0.318 **Fat intake:** g/day, baseline 79 (6), 3 months 85 (6), 6 months 90 (7), 12 months 89 (9) P=0.123	Nocturnal home hemodialysis results in improved appetite and better nutritional status, with potentially positive effect on morbidity and mortality, although this has not been proven in a randomized study.	In the article not mean ±SD were used, but the mean±SE. The SE was rounded. The authors send us the not-rounded numbers and we were able to compute the SE to the SD by using: (SE = SD/ √n)
Spanner, 2003	**nPNA:**NHD patients g/kg/day, (number of patients) baseline 1.03 (0.21) (12), 3 months 1.15 (0.27) (7), 6 months 1.09 (0.30) (8), 9 months 1.14 (0.31) (10), 12 months 1.16 (0.45) (8), CHD patients g/kg/day, (number of patients) baseline 1.02 ± 0.22 (22), 3 months 1.02 (0.16) (21), 6 months 1.05 (0.21) (17), 9 months 1.07 (0.23) (18), 12 months 1.12 (0.30) (20), **Serum albumin:** Nocturnal HD patients g/dL, (number of patients) baseline 3.91 (0.44) (13), 3 months 3.84 (0.46) (12), 6 months 3.92 (0.66) (13), 9 months 3.60 (0.58) [7] (10), 12 months 3.70 (0.52) (9), Control group CHD patients g/dL(number of patients) baseline 3.74 (0.4) (21), 3 months 3.76 (0.45) (19), 6 months 3.77 (0.45) (20), 9 months 3.81 (0.39) (21), 12 months 3.81 (0.41) (20)	**Mean arm muscle area:** Nocturnal HD patients values in cm², (number of patients) baseline 45.83 (11.53) (11), 3 months 45.85 (12.07) (10), 6 months 47.29 (11.66) (8), 9 months 43.33 (10.56) (6), 12 months 43.80 (10.64) (5), **Fat mass:** The daily HD and nocturnal HD groups did not show significant differences, they remained 32% or greater for daily HD patients, and 36% or greater for nocturnal HD patients (no data shown) **BMI:** No significant changes. Mean BMI's for daily HD was ≥27, nocturnal HD ≥23, and control HD patients ≥26 **RBW:** Daily HD patients maintained 100% to 110%% of RBW, nocturnal HD patients showed a significant decline in RBW after 9 months. By that point in the study, their values approached 80% of RBW, indicating a significant decline (no data shown)	**Energy intake:** Food intake remained unchanged, with a trend toward improved kilojoule ingestion in daily HD patient, but not significant P=0.126 (no data shown)	Increased nPNA, serum albumin levels, and arm muscle area suggest that daily HD patients experienced improved nutritional status. (No conclusions of NHD)	This study compared daily HD and NHD with a control group on CHD. The results of the daily HD are not shown in this systematic review.

All figures are shown in mean (± standard deviation), unless stated otherwise.

Abbreviations: PCR: protein catabolic rate, nPCR: normalized protein catabolic rate, CRP: C-reactive protein, BMI: body mass index, ECW: extracellular water, ICW: intracellular water, MUAMC: mid upper arm muscle circumference, % adiposity: fat mass, TBW: total body water, ICW: intracellular water, BCM: body cell mass, nPNA: normalized protein nitrogen appearance, IQR: inter quartile range, RBW: relative body weight.

[1] P < 0.01 compared to baseline.

[2] P < 0.0001 compared to baseline.

[3] Adjusted for age, gender, presence or absence of diabetes, BMI, dialysis duration, center effect and the baseline level of the factor analyzed.

[4] Repeated-measures analyses comparing baseline CHD with 4 and 8 months of NHHD.

[5] The effect size is showed only for parameters that changed significantly in the repeated-measures analysis.

[6] Adjusted means and treatments effects. Adjusted means and treatment effects were estimated under mixed-effects models with adjustment for the baseline level of the outcome and clinical center in the Daily Trail, and the baseline level of the outcome in the Nocturnal Trial (± s.e. or with 95% confidence interval).

[7] P = <0.05 for indicated value versus baseline at that time.
